# Supplementary material for: Empirical methods for controlling false positives and estimating confidence in ChIP-Seq peaks
Source: BMC Bioinformatics. 2008 Dec 5;9:523. doi: 10.1186/1471-2105-9-523 (PMC2628906; doi:10.1186/1471-2105-9-523)
Supplement: Additional File 1 — A variety of html documents from the USeq web site detailing the available applications, their best usage, output file type descriptions, command line menus, etc. [file 1471-2105-9-523-S1.zip › USeqUserGuides/JavaDoc.zip!/JavaDoc/trans/anno/IntersectRegions.html]

IntersectRegions


|  |  |  |
| --- | --- | --- |
| **Overview** \| **Package** \| **Class** \| **Use** \| **Tree** \| **Deprecated** \| **Index** \| **Help** | |  |
| **PREV CLASS**   **NEXT CLASS** | **FRAMES**    **NO FRAMES**     **All Classes** |  |
| SUMMARY: NESTED \| FIELD \| CONSTR \| METHOD | DETAIL: FIELD \| CONSTR \| METHOD |  |


---


## trans.anno Class IntersectRegions

```
java.lang.Object
  trans.anno.IntersectRegions
```

---

``` public class IntersectRegions extends java.lang.Object ```

Performs an intersection analysis on binding region files (chr start stop (optional fraction gc)).

---

**Constructor Summary**

`IntersectRegions(java.lang.String[] arguments)`


| **Method Summary** | |
| --- | --- |
| `static boolean` | `containedWithin(BindingRegion one, BindingRegion two)`             Returns true if two entirely contains one. |
| `static BindingRegion[]` | `fetchChromosomeSpecificBRs(BindingRegion[] sortedBRs, java.lang.String chromosome)`             Given a sorted array of BindingRegion and a chromosome, returns the appropriate BindingRegions. |
| `void` | `intersectBindingRegions()` |
| `void` | `intersectRandomBindingRegions(BindingRegion[] allTrialsOfSingleBR, BindingRegion[] chrOne)`             Assumes the same chromosome |
| `void` | `intersectRandomRegions()`             Increments the hitsPerTrial int[]. |
| `static void` | `main(java.lang.String[] args)` |
| `static BindingRegion[]` | `makeBindingRegions(int[][] regions, java.lang.String chromosome)`             Given an array of start stops and a chromosome, makes BindingRegions. |
| `RandomRegions` | `makeRandomRegions(java.lang.String chromosome)` |
| `static int` | `numberBases(BindingRegion[] brs)` |
| `static BindingRegion[]` | `parseIntervalFile(java.io.File intervalFile)` |
| `static BindingRegion[]` | `parseRegionsFile(java.io.File picksFile)` |
| `static void` | `printDocs()` |
| `void` | `processArgs(java.lang.String[] args)`             This method will process each argument and assign new varibles |
| `double` | `pValue()` |
| `static double[]` | `statBindingRegionArray(BindingRegion[] brs)` |
| `static boolean` | `writeBindingRegions(BindingRegion[] brs, java.io.File file)` |

**Methods inherited from class java.lang.Object**

`equals, getClass, hashCode, notify, notifyAll, toString, wait, wait, wait`

**Constructor Detail**

### IntersectRegions

```
public IntersectRegions(java.lang.String[] arguments)
```


**Method Detail**

### pValue

```
public double pValue()
```

---


### intersectRandomRegions

```
public void intersectRandomRegions()
```

:   Increments the hitsPerTrial int[].

---


### fetchChromosomeSpecificBRs

```
public static BindingRegion[] fetchChromosomeSpecificBRs(BindingRegion[] sortedBRs,
                                                         java.lang.String chromosome)
```

:   Given a sorted array of BindingRegion and a chromosome, returns the appropriate BindingRegions.

---


### makeBindingRegions

```
public static BindingRegion[] makeBindingRegions(int[][] regions,
                                                 java.lang.String chromosome)
```

:   Given an array of start stops and a chromosome, makes BindingRegions.

---


### makeRandomRegions

```
public RandomRegions makeRandomRegions(java.lang.String chromosome)
```

---


### writeBindingRegions

```
public static boolean writeBindingRegions(BindingRegion[] brs,
                                          java.io.File file)
```

---


### intersectRandomBindingRegions

```
public void intersectRandomBindingRegions(BindingRegion[] allTrialsOfSingleBR,
                                          BindingRegion[] chrOne)
```

:   Assumes the same chromosome

---


### containedWithin

```
public static boolean containedWithin(BindingRegion one,
                                      BindingRegion two)
```

:   Returns true if two entirely contains one.

---


### intersectBindingRegions

```
public void intersectBindingRegions()
```

---


### main

```
public static void main(java.lang.String[] args)
```

---


### processArgs

```
public void processArgs(java.lang.String[] args)
```

:   This method will process each argument and assign new varibles

---


### printDocs

```
public static void printDocs()
```

---


### parseRegionsFile

```
public static BindingRegion[] parseRegionsFile(java.io.File picksFile)
```

---


### parseIntervalFile

```
public static BindingRegion[] parseIntervalFile(java.io.File intervalFile)
```

---


### statBindingRegionArray

```
public static double[] statBindingRegionArray(BindingRegion[] brs)
```

:   **Returns:**: double[2]{total bases,median length}.

---


### numberBases

```
public static int numberBases(BindingRegion[] brs)
```

:   **Returns:**: total number of bases in the BindingRegion[], end-start+1.


---


|  |  |  |
| --- | --- | --- |
| **Overview** \| **Package** \| **Class** \| **Use** \| **Tree** \| **Deprecated** \| **Index** \| **Help** | |  |
| **PREV CLASS**   **NEXT CLASS** | **FRAMES**    **NO FRAMES**     **All Classes** |  |
| SUMMARY: NESTED \| FIELD \| CONSTR \| METHOD | DETAIL: FIELD \| CONSTR \| METHOD |  |


---
